# Characterization of Sunflower Oil Extracts from the Lichen *Usnea barbata*

**DOI:** 10.3390/metabo10090353

**Published:** 2020-08-31

**Authors:** Shereen Basiouni, Marwa A. A. Fayed, Reda Tarabees, Mohamed El-Sayed, Ahmed Elkhatam, Klaus-Rainer Töllner, Manfred Hessel, Thomas Geisberger, Claudia Huber, Wolfgang Eisenreich, Awad A. Shehata

**Affiliations:** 1 Clinical Pathology Department, Faculty of Veterinary Medicine, Benha University, Moshtohor, Toukh 13736, Egypt; awad.shehata@vet.usc.edu.eg; 2 Pharmacognosy Department, Faculty of Pharmacy, University of Sadat City, Sadat 32897, Egypt; marwa.fayed@fop.usc.edu.eg; 3 Institute for Bacteriology and Mycology, Faculty of Veterinary Medicine, University of Sadat City, Sadat 32897, Egypt; reda.tarabees@vet.usc.edu.eg (R.T.); mohamed.sabry@vet.usc.edu.eg (M.E.-S.); 4 Department for Parasitology, Faculty of Veterinary Medicine, University of Sadat City, Sadat 32897, Egypt; ahmed.osman@vet.usc.edu.eg; 5 Research and Development Section, PerNaturam GmbH, An der Trift 8, 56290 Gödenroth, Germany; toellner@pernaturam.de (K.-R.T.); hessel@pernaturam.de (M.H.); 6 Chair of Biochemistry, Department of Chemistry, Technical University Munich, Lichtenbergstraße 4, 85748 Garching, Germany; thomas.geisberger@tum.de (T.G.); claudia.huber@tum.de (C.H.); 7 Avian and Rabbit Diseases Department, Faculty of Veterinary Medicine, University of Sadat City, Sadat 32897, Egypt

**Keywords:** usnic acid, *Usnea barbata*, *Enterococcus faecalis*, enterococcosis, natural antimicrobial, multidrug resistant bacteria, cytotoxicity, phenolics, flavonoids

## Abstract

The increasing global emergence of multidrug resistant (MDR) pathogens is categorized as one of the most important health problems. Therefore, the discovery of novel antimicrobials is of the utmost importance. Lichens provide a rich source of natural products including unique polyketides and polyphenols. Many of them display pharmaceutical benefits. The aim of this study was directed towards the characterization of sunflower oil extracts from the fruticose lichen, *Usnea barbata*. The concentration of the major polyketide, usnic acid, was 1.6 mg/mL extract as determined by NMR analysis of the crude mixture corresponding to 80 mg per g of the dried lichen. The total phenolics and flavonoids were determined by photometric assays as 4.4 mg/mL (gallic acid equivalent) and 0.27 mg/mL (rutin equivalent) corresponding to 220 mg/g and 13.7 mg/g lichen, respectively. Gram-positive (e.g., *Enterococcus faecalis*) and Gram-negative bacteria, as well as clinical isolates of infected chickens were sensitive against these extracts as determined by agar diffusion tests. Most of these activities increased in the presence of zinc salts. The data suggest the potential usage of *U. barbata* extracts as natural additives and mild antibiotics in animal husbandry, especially against enterococcosis in poultry.

## 1. Introduction

The emergence of multidrug resistant (MDR) pathogenic bacteria poses a worsening and general health problem [1]. This phenomenon was mainly caused by excessive use of antimicrobials as therapeutics and preventive tools in the animal industry and large-scale poultry operations. This challenge has led to a constant search for the most suitable alternatives for in-feed antimicrobials [2,3]. The WHO has recently rated the phenomenon of MDR among the most important problems threatening human health, as there are large gaps in the existing surveillance of antimicrobial resistance in many parts of the world [4].

In large poultry operations, antimicrobials have been extensively used as therapeutics and growth promoters [5,6,7]. However, the indiscriminate usage of antimicrobials and the increasing levels of antibiotic residuals in animal products, particularly in meat and poultry products, can have serious effects on human health [8,9]. Among the most important MDR bacteria, the “ESKAPE” group encompassing *Enterococcus faecium*, *Staphylococcus aureus*, *Klebsiella pneumoniae*, *Acinetobacter baumannii*, *Pseudomonas aeruginosa*, and *Enterobacter* strains can escape the biocidal action of most antimicrobial agents [10]. In the poultry industry, a steadily increasing level of MDR bacteria has also been reported, for example of *Escherichia coli* [11], *Campylobacter* [12,13,14], *Salmonella* [15,16], and methicillin-resistant *S. aureus* (MRSA) [17,18].

Recent achievements in phytochemical and phytopharmacological sciences have facilitated clarification of the composition and biological activities of many medicinal plants (for an example, see [19]). Plant extracts and essential oils have been used since ancient times for the treatment of various diseases and have also been established in modern medicine mainly because of their availability and lower toxicity as compared with related synthetic drugs (for an example, see [20]).

Lichens are worldwide symbiotic associations of fungi with microalgae or cyanobacteria [21], and, next to plants, they represent an important source of biologically active natural compounds [22,23]. For now, more than 800 lichen-specific secondary metabolites have been described and many of them exhibit beneficial bioactivities. These unique secondary metabolites usually represent 0.1–10% and sometimes even 30% of the dry vegetable mass of lichens, thus, providing a rich source for these natural compounds.

Due to their bioactivities, lichens have been used for centuries in traditional medicine. Nowadays, it is known that many of their unique compounds exhibit antimicrobial, antitumor, antimutagenic, antifungal, or antiviral effects (for some examples, see [24,25,26,27,28]), and therefore lichens are considered to be a treasure trove for natural compounds which can be further developed for applications in modern medicine. In the poultry industry, in vitro studies have shown that the crude polysaccharide fraction from *Parmelia perlata* has antiviral activities against RNA viruses including yellow fever virus, poliovirus, and infectious bursal disease virus [29]. Notably, however, it has also been shown that the antimicrobial activities of lichen extracts significantly varied according to the species and the method of extraction [30,31]. Therefore, careful quality control of lichen products is crucial prior to their usage.

**Figure 1:**
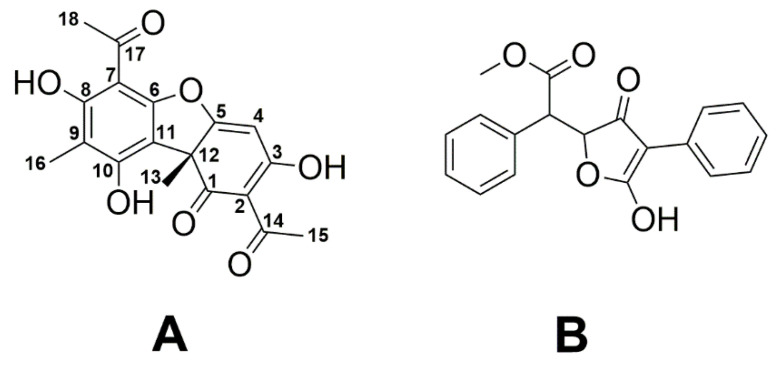
Chemical structures of (+)-usnic acid (**A**) and vulpinic acid (**B**).

*Usnea* is a widespread and well-known genus of lichens found in many countries. Indeed, *Usnea barbata* commonly known as “old man’s beard”, “beard lichen” or “treemoss” has been used in traditional medicine for the treatment of diarrhea, ulcers, urinary infections, tuberculosis, pneumonia, stomachache, and cattle fungal disease [32]. In about 500 *Usnea* species including *U. barbata*, the polyketide usnic acid (UA), a dibenzofuran derivative existing in two enantiomeric forms, represents the most important secondary metabolite [26,33,34,35]. More specifically, (+)-UA (Figure 1) is the predominant form in *Usnea* species [36,37].

UA is one of the most extensively studied lichen metabolites with controversial results related to its benefits, apparently depending on the method of extraction and the lichen species [38,39]. The polyketide has been claimed to serve weight loss in humans, although hepatotoxic effects were also reported [40,41,42]. In addition, UA exhibits antibacterial, antiviral, antifungal, antiprotozoal, insecticidal, anti-inflammatory, and cytotoxic activities (reviewed in [26]). Notably, UA also inhibits the growth of Gram-positive bacteria including *S. aureus*, *Enterococcus faecalis*, *E. faecium*, and some anaerobic species similar to vulpinic acid, another well-known antimicrobial lichen metabolite (Figure 1) [43]. It has also been reported that UA is potent for the treatment of trichomoniasis in pigeons [44].

Previous reports have shown that some salts of UA were more efficient than free acid. For example, sodium UA displayed a superior acaricidal activity [45]. Under aqueous conditions, usnates are more soluble than the free acid with no toxicity reported [46]. As an example, zinc salts of UA have been used as adjuvants for the effective treatment of human papillomavirus with only minor side effects [47].

Herein, we report a simple and low-cost method for extracting *U. barbata* using sunflower oil in the cold. We show that this extract is characterized by high amounts of UA, polyphenols, and flavonoids per gram of lichen as compared with commercial CO_2_ extracts of the same lichen. The sunflower oil extract and its zinc salt very efficiently inhibited the growth especially of *E. faecalis* and displayed moderate antimicrobial activities also against other Gram-negative and Gram-positive reference pathogens, as well as against bacterial isolates (e.g., *E. faecalis*) from infected chickens.

## 2. Results

### 2.1. NMR-Based Determination of Usnic Acid in Oily Extracts of U. barbata

**Figure 2:**
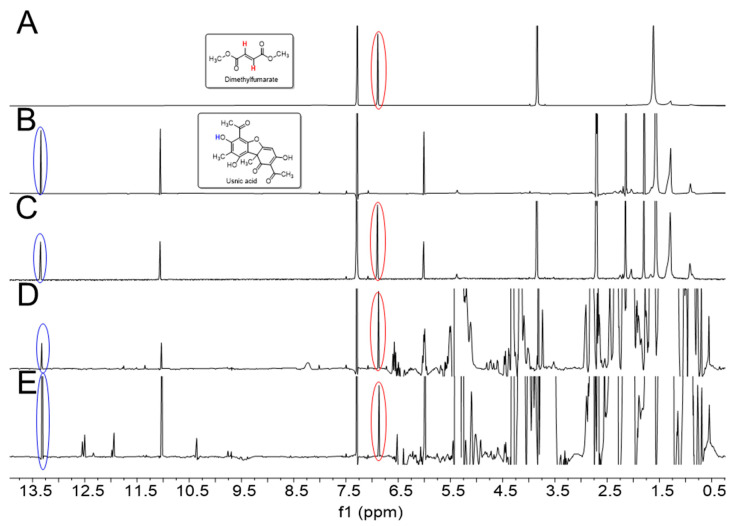
(**A**) ^1^H-NMR spectrum of dimethyl fumarate (DMFum). The singlet signal (6.89 ppm) of the alkene moiety is indicated by the red circle; (**B**) ^1^H-NMR spectrum of usnic acid (UA). The well separated singlet signal of the OH proton at C-8 (13.33 ppm) is indicated by the blue circle; (**C**) ^1^H-NMR spectrum of a 1:1 molar mixture (DMFum/UA), showing the expected integral ratio of the DMFum and UA signals of approximately 2:1; (**D**) ^1^H-NMR spectrum of *U. barbata* extract using sunflower oil as the extractant; (**E**) ^1^H-NMR spectrum of a commercial *U. barbata* CO_2_ extract (Flavex^®^). All spectra (except for (**B**)) are scaled to the same height of the DMFum signals.

For a rapid identification and quantification of UA in crude extracts of *U. barbata*, we used ^1^H-NMR spectroscopy of the mixture that relied on the detection of specific and well separated signals for UA in the concurrent presence of abundant lipid signals from the extractant (Figure 2D). The NMR signals of UA were unequivocally assigned by two-dimensional NMR experiments, as reported earlier [48]. It turned out that the downfield shifted ^1^H singlet at 13.33 ppm due to the OH group at C-8 of UA (for carbon numbering, see Figure 1) was most useful for quantifying the polyketide in the complex mixture (cf. Figure 2B for a ^1^H-NMR spectrum of pure UA). In our procedure, the intensity of this signal was referenced to the intensity of the ^1^H-NMR signal at 6.89 ppm for the -CH=CH-protons of the internal reference, dimethyl fumarate (DMFum), which was added to the NMR sample at a known concentration (cf. Figure 2A for a 1H-NMR spectrum of pure DMFum).

The spectrum of a 1:1 molar mixture of DMFum/UA displayed in Figure 2C shows the validity of the quantitative approach. The integral ratio of the signals due to DMFum and UA in this spectrum was determined as 2:1.02, quite accurately reflecting the relative numbers of protons for these signals provided by the respective compounds in equimolar amounts, i.e., two for DMFum and one for UA. As another control, Figure 2E shows the NMR spectrum of Flavex^®^, a commercial CO_2_ extract of *U. barbata* containing about 38 mg UA per mL, according to the manufacturer’s information. Here, the spectral region from 0.5 to 5.5 ppm is characterized by intense signals due to lipids (e.g., sunflower oil and polyglyceryl-3-palmitic acid) which are used by the manufacturer to dissolve the CO_2_ extract. Even in this complex mixture, the signals for the OH protons at position 3 (11.05 ppm) and 8 (13.33 ppm) of UA turned out to be well separated and the OH-8 signal could again be subjected to quantitative analysis, as described above. Using Equation (2) for calculation (see Materials and Methods) revealed a concentration of 31.5 ± 1.2 mg UA per ml Flavex^®^ sample which was in the expected range.

The ^1^H-NMR spectrum of the crude sunflower oil extract of *U. barbata* is shown in Figure 2D. The spectrum was also dominated by huge signals due to the lipids from the sunflower oil, but the OH signals of UA were again well resolved allowing to quantify the content of UA. The detected normalized integral values I (UA) of OH at C-8 for UA in three replicates (A–C), each normalized to the integral of the internal standard (DMFum = 1.00), were as follows: sample A, I (UA) = 0.36; sample B, I (UA) = 0.34; and sample C, I (UA) = 0.33. Using Equation (2), the concentration of UA was determined as 1.6 ± 0.09 mg per mL extract. This corresponds to an amount of 80.4 ± 3.5 mg UA/g dry lichen.

### 2.2. Estimation of Total Phenolic and Flavonoid Contents

The total phenolic content in the extract was estimated by a colorimetric assay using gallic acid as a reference. In the concentration range of 5–50 µg gallic acid per mL, the assay had a linear response with a regression coefficient (R^2^) = 0.9986, a slope (m) = 0.0027 and an intercept = −0.0143. Using this equation, the total phenolics were determined as 4.4 mg ± 1.4 mg/mL extract corresponding to 220 mg/g dried lichen.

The total flavonoid content of the extract was measured using the aluminum chloride colorimetric assay using rutin as a reference. The rutin solution (5–100 µg/mL) conformed to Beer’s Law at 510 nm with a regression co-efficient (R^2^) = 0.9946. The plot had a slope (m) = 0.0011 and an intercept = 0.0131. On the basis of this, the total flavonoids were determined as 0.27 mg ± 0.11 mg/mL extract, corresponding to 13.7 mg/g dried lichen.

### 2.3. Antimicrobial Activity

**Figure 3:**
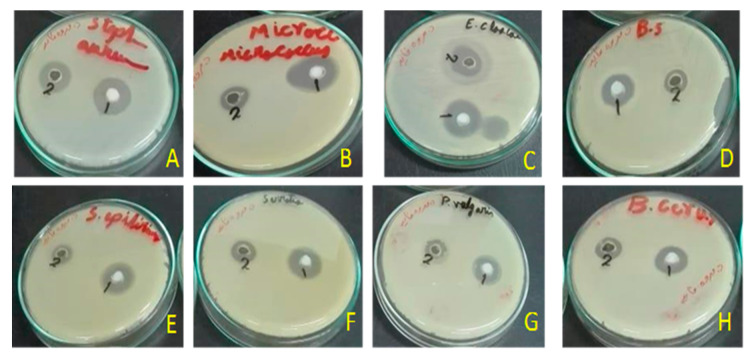
Antimicrobial activity of the zinc salt of extract (1) and the total sunflower-oil extract (2) of *U. barbata*. (**A**) *Staphylococcus aureus* (RCMB010010); (**B**) *Micrococcus* sp. (RCMB 028); (**C**) *Enterobacter cloacae* (RCMB 001, ATCC 23355); (**D**) *Bacillus subtilis* (RCMB 015); (**E**) *Staphylococcus epidermidis* (RCMB 009); (**F**) *Micrococcus* spp (RCMB 028); (**G**) *Proteus vulgaris* (RCMB 004, ATCC 13315); (**H**) *Bacillus cereus* (RCMB 027).

**Figure 4:**
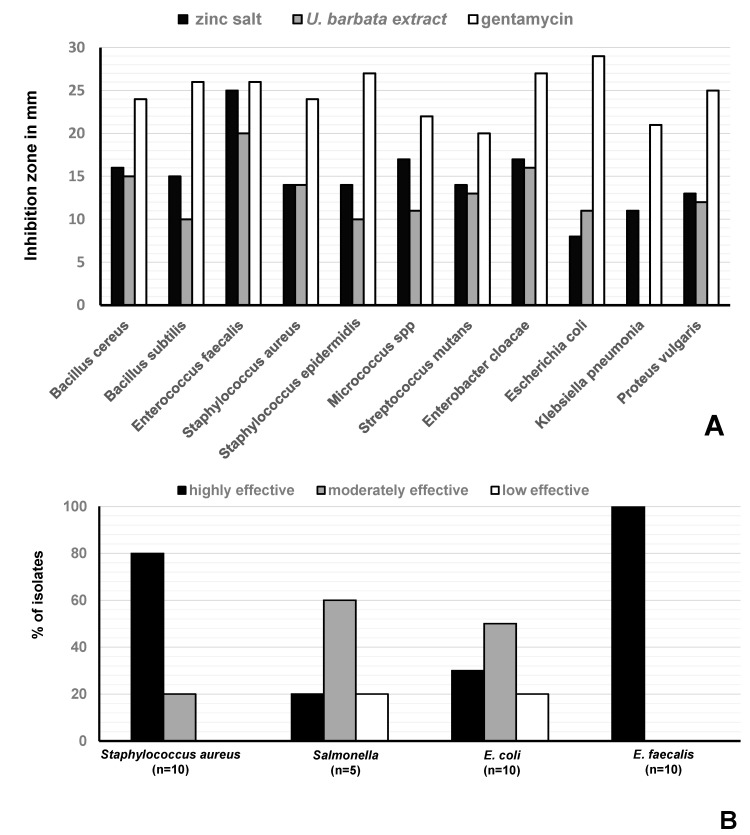
Antimicrobial activity of *U. barbata* preparations. (**A**) Size of the inhibition zone of the sunflower-oil *U. barbata* extract and its zinc salt on Gram-positive and Gram-negative reference strains; (**B**) In vitro effect of the salt on *S. aureus* (*n* = 10), *Salmonella* spp (*n* = 5), *E. coli* (*n* = 10), and *E. faecalis* (*n* = 10) strains isolated from chickens. Not effective (very small inhibition zone) ≤8 mm, low effective (small inhibition zone) = 8–13, moderately effective (medium inhibition zone) = 14–19 mm, and highly effective (large inhibition zone) ≥20 mm. Sunflower oil was used as negative control and did not cause a zone of inhibition.

Disc diffusion tests revealed antimicrobial activities against Gram-positive and Gram-negative bacteria (Figure 3). In these tests, we used 50 µL of the *U. barbata* sunflower oil extract which was 1:1 (*v*/*v*) diluted in DMSO (corresponding to ca. 40 µg UA per assay) or the respective amounts of the precipitated zinc salt. Most importantly, both samples were highly effective (inhibition zone ≥ 20 mm) against *Enterococcus faecalis* (Figure 4A). Here, inhibition due to the reference antibiotic, gentamycin, was comparable when present at approximately equimolar amounts of UA. Moreover, the extract exhibited a moderate antimicrobial activity (inhibition zone = 14–19 mm) against *Bacillus cereus*, *S. aureus*, *Streptococcus mutans*, and *Enterobacter cloacae*. Lower activity (inhibition zone = 8–13 mm) was observed for *B. subtilis*, *S. epidermidis*, *Micrococcus* spp, *E. coli*, and *Proteus vulgaris* (Figure 4A). The zinc salt precipitate was more effective (with the exception of *E. coli*), but still only moderately active (inhibition zone = 14–19 mm) against these bacteria (Figure 4A). The zinc precipitate and the extract, both, did not exhibit any antifungal effects against *Aspergillus fumigatus* and *Candida albicans*.

The zinc salt exhibited different antibacterial effects on bacterial strains isolated from poultry, i.e., against *S. aureus* strains (*n* = 10), *Salmonella* strains (*n* = 5), *E. faecalis* strains (*n* = 10), and *E. coli* strains (*n* = 10) with *n* = number of isolates (Figure 4B, Table 1). It was highly effective (inhibition zone ≥ 20 mm) against all (100%) tested *E. faecalis* strains. It was moderately effective (inhibition zone = 14–19 mm) and less effective (inhibition zone = 8–13 mm) on 8/10 (80%), and 2/10 (20%) of the tested *S. aureus* strains, respectively. It was highly effective, moderately effective, and less effective against 1/5 (20%), 3/5 (60%), and 1/5 (30%) of the tested *Salmonella* spp, respectively. Finally, it was highly effective, moderately effective, and less effective against 30%, 50%, and 20% of the tested *E. coli* strains, respectively (Figure 4B).

### 2.4. Cytotoxicity

Two concentrations of the extract, i.e., 10 or 100 µL of the extract in 1 mL DMEM, corresponding to 16 or 160 µg UA per mL of the resulting mixtures, respectively, and respective amounts of its zinc salt precipitate, were analyzed using cell viability assays with MCF-7 and HEPG-2 cells (Table 2). On the one hand, treatment of MCF-7 and HEPG-2 with *U. barbata* extract (10 µL/mL) for 24 h induced 97 and 93% viability, respectively. However, the higher concentration (100 µL/mL) induced only 31 and 46% viability, respectively. On the other hand, treatment of MCF-7 and HEPG-2 with the prepared zinc salt of the *U. barbata* extract at concentrations of 10 µL/mL, induced 90 and 98% viability, respectively. At higher concentrations of the zinc salt (100 µL/mL), the viability decreased to 44 and 6%, respectively.

## 3. Discussion

The emergence of MDR bacterial strains in the human food chain is still a main concern for public health and food safety authorities worldwide. The search for proper alternatives that fulfill the demands for antimicrobials without developing resistance is the topic of many research studies. Here, we have focused on the well-known antibiotic properties of extracts of the lichen *U. barbata* containing the polyketide UA in about 2% of the dried lichen, according to literature [39,52]. Most previous studies with *U. barbata* used methanol or ethyl acetate as solvents [53] or supercritical CO_2_ for the extraction. For the analysis of UA and other components, high performance liquid chromatography (HPLC) [54,55], and ultra-high-performance liquid chromatography (UHPLC) were typically used [56].

In the present study, *U. barbata* was extracted by an alternative mild procedure, in which the lichen material was treated for three months by cold sunflower oil. For the detection of UA, we exploited quantitative NMR spectroscopy of the crude extract. It turned out that NMR spectroscopy was appropriate to assess the target compound in the crude metabolite mixture without any prior chromatographic procedures.

Using the protocol described in the Materials and Method section, we could unequivocally quantify the amounts of UA as 1.6 ± 0.09 mg per mL extract corresponding to 80.4 ± 3 mg per g lichen (i.e., about 8% of the dried lichen material). In the commercial *U. barbata* extract using supercritical CO_2_ as extractant (Flavex^®^ Naturextrakte GmbH, Rehlingen-Siersburg, Germany), about 32 mg UA per mL extract were determined, corresponding to about 13 mg UA per g lichen, assuming that 2–3 kg of lichen were used for the preparation of 1 L Flavex^®^ extract according to the manufacturer. On this basis, the yields of UA are significantly higher when extracting *U. barbata* with sunflower oil in the cold for a long period. It should also be noted that the purity of UA seems to be higher in the sunflower oil extract when comparing the high-field regions (>9 ppm) of the NMR spectra (compare Figure 2D,E). These findings probably reflect that the yield of UA is correlated with the solubility of this compound in the solvent used for extraction [38,57,58], the temperature, and the time assigned to the operation [34]. However, the UA content in extracted lichens certainly also depends on additional more complex parameters including the geographical origin of the lichen and the time of sampling.

The antimicrobial and cytotoxic activities of UA have been the topics of many previous studies for more than 50 years [26,33,34]. The efficacy of UA against *S. aureus*, *E. faecium*, and *E. faecalis* has also been reported earlier [43,59] and it has been shown that UA significantly inhibited the formation of bacterial biofilms on polymer surfaces [60]. In the present study, the antimicrobial and antimycotic activities of the *U. barbata* sunflower-oil extract and its zinc salt precipitate were assessed against several Gram-positive and Gram-negative bacteria, as well as against two fungal strains and compared with reference antibiotic and antimycotic agents (gentamycin and ketoconazole, respectively). Interestingly, no antimycotic activities against the tested fungi could be observed in contrast to literature data [55]. However, it should be noted that the antimycotic effect on only two available strains was analyzed in our study.

In sharp contrast, significant inhibition of bacterial growth could be observed for all bacteria under study. Compared to the high antimicrobial effect of similar amounts of the reference antibiotic gentamycin (inhibition zone ≥ 20 mm), the *U. barbata* sunflower-oil extract and its zinc salt exhibited lower, but moderate antibacterial effects (inhibition zone = 14–19 mm) against several Gram-positive and Gram-negative bacteria including *S. aureus* and *E. cloacae*, respectively (for a notable exception, see below).

In addition, the antimicrobial activities of the extract and its zinc salt were evaluated against bacterial isolates from infected chicken. Concerning *S. aureus*, *Salmonella*, and *E. coli* isolates, the inhibition appeared diverse from no to moderate inhibition. On this basis, no generalized assertion about the antibacterial activities and mechanisms of the extract or its zinc salt against these pathogenic species isolated from poultry could be drawn.

However, high activities (inhibition zone ≥ 20 mm) were observed against the reference strain of *E. faecalis* and against all (10/10) clinical isolates of *E. faecalis* from chickens. This finding is of special importance since this Gram-positive bacterium is known to cause several clinical findings in poultry such as first-week mortality in chicks [61], amyloid arthropathy in layers [62,63], and pulmonary hypertension syndrome in broilers [64], as well as hepatic granulomas in turkey poults [65]. Moreover, poultry could be a source of MDR enterococci [66,67] that could result in nosocomial infections in humans such as urinary tract infections, bacteraemia or endocarditis [68,69,70,71].

Our results also indicate that most antimicrobial activities of the extract could be increased in the presence of zinc salts. This was consistent with the finding that sodium salts of UA exhibited potent antimicrobial activities against vancomycin-resistant enterococci and MRSA [72]. Further investigations are certainly needed to optimize a most effective salt combination and, also to verify the molecular mechanisms of the observed bioactivities.

In addition to the antimicrobial effects, cytotoxic activities of UA and UA derivatives are known from previous studies. It has been shown that both UA enantiomers have cytotoxic and apoptotic activities on Chinese hamster lung fibroblast-like (V79) and human lung carcinoma epithelial-like (A549) cell lines [73]. Moreover, it has been demonstrated that UA derivatives have cytotoxic and apoptotic activities on a mouse lymphocytic leukemia cell line (L1210) and Chinese hamster ovary (CHO) cell lines [74]. Furthermore, it has been reported that UA derivatives inhibited the growth of human hepatoma HepG2 cells [75].

Zinc ions are known to protect HEPG2 against oxidative stress and DNA damage induced by mycotoxins increasing cell viability [76]. In our study, a significant cytotoxicity (*p* < 0.0001) effect of the zinc precipitate of the lichen extract on HEPG2 at high doses (100 µL/mL) was found as compared with the total extract. This could be attributed to the zinc ions of this precipitate. In line with this hypothesis, recently, it was found that zinc accumulation induces oxidative stress and subsequent apoptosis of HEPG2 due to mitochondrial dysfunction [77].

In the presence of high amounts of polyphenols and flavonoids, probably serving as antioxidant, radical scavenging compounds in *U. barbata* extracts, the cytotoxic effects of UA could be modulated. Therefore, in vitro cytotoxic tests using the sulphorhodamin B (SRB) assay were carried out showing that both the *U. barbata* sunflower oil extract and its zinc salt significantly inhibited the proliferation of MCF-7 and HEPG-2 cells in a dose-dependent manner. Interestingly, the effect of the zinc salt was, again, more potent as compared with the cytotoxic activities of the whole extract. Further in vivo investigations are required to confirm these preliminary data and to ensure the activity and safety of these compounds as potential cytotoxic agents, especially at high concentrations.

## 4. Conclusions

In the present study, we established a rapid NMR method to directly detect and quantify UA incrude oily extracts of *Usnea*. It appears that higher yields of UA per g of dry lichen can be obtained when using cold sunflower oil for a long period as extractant as compared with extracts using supercritical CO_2_. The oil extract and its zinc salt exhibited high antimicrobial activities against *E. faecalis* and moderate activities against other Gram-positive or Gram-negative bacteria. Especially, the high activity observed against *E. faecalis* and 10 of 10 clinical isolates of this pathogen from infected chickens is promising and underlines the potential usage of *Usnea* sunflower oil extracts or its zinc salt as natural feed additives for poultry, particularly to treat or prevent enterococcosis. Nevertheless, antibiograms could still be required to determine the doses and specificities of *Usnea* extracts before beginning a potential therapy of infected chickens or other animals.

## 5. Materials and Methods

### 5.1. Materials and Extraction of U. barbata

**Figure 5:**
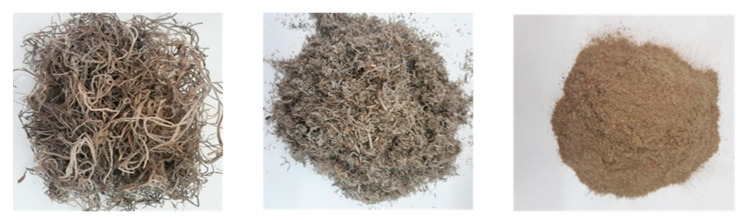
The lichen *U. barbata* used in this study. Whole (**left**); Macerated (**middle**); Powder (**right**).

*U. barbata* raw materials, originated from Mexico, were obtained from Heinrich Klenk GmbH, Schwebheim, Germany (Figure 5). Air-dried and cleaned lichen materials were ground in a household blender. The lichen samples (20 g) were separately blended with 1 L of Bio-sunflower oil (OPW Ingredients GmbH, Niederkrüchten, Germany) at room temperature, in the dark, for 3 months, and then filtered. This extract was used to determine the concentrations of UA, total phenolics and flavonoids, as well as for screening of antimicrobial and cytotoxicity activities.

A commercial *U. barbata* CO_2_ extract dissolved in oil (Flavex^®^ Naturextrakte GmbH, Rehlingen-Siersburg, Germany) was used as a positive control for UA determination. According to the producer, 2–3 kg of *U. barbata* raw material, originated from Macedonia, were used to produce 1 L extract containing 38–42 mg/mL (3.8–4.2%) UA. In these samples, the *U. barbata* CO_2_ extract is dissolved in 70% sunflower oil and 25% polyglyceryl-3-palmitate. Sunflower oil (OPW Ingredients GmbH, Niederkrüchten, Germany) was used as a placebo control.

### 5.2. Preparation of a Zinc Salt Precipitate

A total of 25 g of the total extract in 250 mL of water was heated at 50 °C. A volume of 5 mL of 1 M NaOH (0.005 mol) was added and 0.4 g (0.005 mol) of ZnO in 40 mL of water was added, and the resulting suspension was stirred, for 30 min, at 50 °C. Then, the pH was adjusted to 7.3 by addition of aqueous HCl. The resulting precipitate was filtered at reduced pressured and washed with dH_2_O to yield the zinc salt of UA and other acids [78].

### 5.3. Quantification of UA by NMR

For identification and quantification of UA in the extract, three replicates (A–C) of the sunflower oil extract were analyzed. For each replicate, a volume of 100 µL was transferred into a standard 5 mm NMR tube which was, then, filled with 100 µL of a solution of fumaric acid dimethylester (DMFum) in CDCl_3_ (1 mg/mL) and 360 µL of CDCl_3_. This mixture was analyzed by ^1^H-NMR spectroscopy using a Bruker Avance III spectrometer operating at 500 MHz and a temperature of 27 °C. For typical measurements, 256 scans were collected. The acquisition time was 3.2 s, the relaxation delay was 10 s, and the data size was 32 k. Control measurements with a relaxation delay of 30 s resulted in the same ratios of UA and DMFum integrals. Before Fourier transformation, the FIDs were multiplied with a mild Gaussian curve (corresponding to a lb value of −1 and a gf value of 0.01, using the MestreNova software). The phases and baselines were manually corrected for each spectrum. For a typical spectrum of the extract and an UA reference (0.1 mg in the sample), see Figure 2B,D, respectively. The observed integrals for the well-resolved and well-separated signals at 6.89 ppm (-CH=CH- protons of DMFum) and at 13.33 ppm (OH at C-8 of UA) [43], then, served to quantify the polyketide. To calculate the amount of UA, Equation (1) was used:

(1) $$m\left(UA\right)=\frac{I\left(UA\right)}{I\left(DMFum\right)}*\frac{N\left(DMFum\right)}{N\left(UA\right)}*\frac{M\left(UA\right)}{M\left(DMFum\right)}*m\left(DMFum\right)$$

*m* (*UA*) = mass of *UA*; *m* (*DMFum*) = mass of dimethylfumarate; *I* (*UA*) = NMR integral for *UA*; *I* (*DMFum*) = NMR integral for dimethylfumarate; *N* (*UA*) = proton count for the NMR signal of *UA*; *N* (*DMFum*) = proton count for the NMR signal of dimethylfumarate; *M* (*UA*) = molar mass of *UA*; *M* (*DMFum*) = molar mass of dimethylfumarate

Equation (1) was simplified to Equation (2):

(2) m(UA) in µg=I(UA)1∗21∗344 gmol144 gmol∗100 µg

Equation (2) already contains the value of the NMR integral of *DMFum* normalized to 1.00, the number of protons causing the respective signals (*DMFum* = 2 and *UA* = 1), and the molar masses of the two compounds (*DMFum* = 144 g/mol and *UA* = 344 g/mol), and the mass of *DMFum* added as the internal reference (100 µg).

As a control, we calculated the recovery of *UA* in a reference mixture, where we added 172 µL of a standard *UA* solution (1 mg/mL in CDCl_3_) corresponding to 172 µg of *UA* and 72 µL of a standard solution of *DMFum* (1 mg/mL in CDCl_3_). On the basis of the 1H-NMR analysis and using Equation (2), we calculated an amount of 175 µg of *UA* in this sample (recovery, 102%).

### 5.4. Quantification of Total Phenolics and Flavonoid

The total phenolic content of the total extract was determined by the Folin–Ciocalteu method [79]. Gallic acid was used as a standard, and the total phenolic content was expressed in terms of gallic acid equivalents (GAE). The absorbance was monitored at 725 nm. The total flavonoid content was determined by a colorimetric assay based on the formation of an aluminum chloride complex. Rutin was used as a standard, and the flavonoid content was determined in terms of rutin equivalents (RE). The absorbance of the reaction mixture was measured at 510 nm [80]. All procedures were performed in 6 replicates. The phenolic and flavonoid contents of the extract were expressed as mg GAE or RE per gram of dried sample.

### 5.5. Antibiograms

The antimicrobial activities of the total extract and the prepared zinc salt of the extract were evaluated using the well-established agar diffusion method [81] against different reference microorganisms including Gram-positive bacteria (*Bacillus cereus* (RCMB 027), *Bacillus subtilis* (RCMB 015), *Enterococcus faecalis* (ATCC 29212), *Staphylococcus aureus* (RCMB010010), *Staphylococcus epidermidis* (RCMB 009), *Micrococcus* spp (RCMB 028), *Streptococcus mutans* (RCMB 017, and ATCC 25175)), Gram-negative bacteria (*Enterobacter cloacae* (RCMB 001, ATCC 23355), *Escherichia coli* (RCMB 010052, ATCC 25955), *Klebsiella pneumonia* (RCMB 003, ATCC 13883), and *Proteus vulgaris* (RCMB 004, ATCC 13315)), and yeasts and fungi (*Candida albicans* (RCMB 005003, ATCC 10231), and *Aspergillus fumigatus* (RCMB 002002)).

Additionally, the antimicrobial activity was assessed for Gram-positive and Gram-negative bacterial species isolated from poultry such as *Staphylococcus aureus* (*n* = 10), *Salmonella* (*n* = 5), *Enterococcus faecalis* (*n* = 10), and *E. coli* (*n* = 10) (*n* = number of isolates). The cultural origin of these chicken isolates and their culture conditions are summarized in Table 1.

The inoculum suspensions were prepared from colonies grown overnight on an agar plate and inoculated into Mueller–Hinton broth (fungi using malt broth). The extract or the salt precipitate was dissolved in dimethyl sulfoxide (DMSO) (20 mL extract/20 mL DMSO). From this solution, 50 µL corresponding to ca. 40 µg of UA were dropped onto the plate. The inhibition zone was measured around each well after 24 h, at 37 °C. Controls using DMSO were adequately done. The activities were evaluated according to the size of inhibition zone as described by Bismarck et al. [82] as follows: not effective ≤8 mm, low effective = 8–13 mm, moderately effective = 14–19 mm, and highly effective ≥20 mm. Readymade antibiotic discs containing gentamycin (10 µg, Oxoid, Wesel, Germany) or ketoconazole (15 µg, Rosco Diagnostica, Taastrup, Denmark) were used as positive controls for antibacterial and antifungal activities, respectively.

### 5.6. Cytotoxicity

To assess cytotoxicity of the extract and its salt precipitate, the human breast cell line MCF-7 and hepatoblastoma cell line HEPG2 (Leibniz Institute DSMZ - German Collection of Microorganisms and Cell Cultures, Braunschweig Germany) were used. Cells were maintained in DMEM media supplemented with 100 mg/mL of streptomycin, 100 units/mL of penicillin, and 10% of heat-inactivated fetal bovine serum in humidified, 5% (*v*/*v*) CO_2_ atmosphere at 37 °C. Cell viability was assessed by the sulforhodamine B (SRB) assay. Briefly, aliquots of 100 µL cell suspension (5 × 10^3^ cells) filled into 96-well plates were incubated in complete media for 24 h. Cells were treated with another aliquot of 100 µL medium containing the extract and its salt under study at final concentrations of 10 and 100 µg/mL, respectively. After 72 h of exposure, cells were fixed by replacing media with 150 µL of 10% TCA and incubated at 4 °C for 1 h. The TCA solution was removed, and the cells were washed 5 times with distilled water. Aliquots of 70 µL SRB solution (0.4%, *w*/*v*) were added and incubated in a dark place at room temperature for 10 min. Plates were washed 3 times with 1% acetic acid and allowed to air-dry overnight. Then, 150 µL of TRIS (10 mM) were added to dissolve protein-bound SRB stain; the absorbance was measured at 540 nm using a BMG LABTECH^®^-FLUOstar Omega microplate reader (Ortenberg, Germany).

### 5.7. Statistical Analysis

Data of cytotoxicity are shown as means with standard errors (SEM). Unpaired Students *t* test was used to identify significant differences between means. The statistical analysis was carried out with GraphPad Prism 4 (GaphPad Software, La Jolla, CA, USA). In all cases, *p* < 0.05 was assumed to indicate significant differences.

## Figures and Tables

**Table 1:** Target strains used in this study, their origin and culture conditions.

Bacterial Isolates	Reference/Lab. No.	Media Used for Antibiogram	History of Isolates	References
*Staphylococcus aureus*	11/12	Mueller–Hinton agar	Commercial broilers showing arthritis	[49]
*Staphylococcus* *aureus 2*	Staph-2/20	Mueller–Hinton agar	Isolated from one-week old commercial broilers chicks allocated in 10 different farms suffering from arthritis and omphalitis. Isolation was done on blood agar (Fluka, Steinheim, Germany) and identified using biochemical tests (data not shown)	this study
*Staphylococcus aureus -3*	Staph-3/20	Mueller–Hinton agar		this study
*Staphylococcus aureus -4*	Staph-4/20	Mueller–Hinton agar		this study
*Staphylococcus aureus -5*	Staph-5/20	Mueller–Hinton agar		this study
*Staphylococcus aureus -6*	Staph-6/20	Mueller–Hinton agar		this study
*Staphylococcus aureus -7*	Staph-7/20	Mueller–Hinton agar		this study
*Staphylococcus aureus -8*	Staph-8/20	Mueller–Hinton agar		this study
*Staphylococcus aureus -9*	Staph-9/20	Mueller–Hinton agar		this study
*Staphylococcus aureus -10*	Staph-10/20	Mueller–Hinton agar		this study
*Salmonella* Gallinarum	Z34/11	Caso-agar	Commercial broiler chickens showing diarrhea, pasty vent, and high mortality rates	[50]
*Salmonella Enteritidis-1*	SE-1	Caso-agar	Commercial broiler chicken	[51]
*Salmonella Enteritidis-2*	SE-2	Caso-agar	Commercial broiler chicken showing diarrhea and mortalities	[15]
*Salmonella Typhimurium-1*	ST-1	Caso-agar		[15]
*Salmonella Typhimurium-2*	ST-2	Caso-agar		[15]
*E. coli-1*	E-1/20	Mueller–Hinton agar	Isolated from visceral organs (livers, lungs, hearts, and spleens) collected from diseased and freshly dead broiler chickens in 10 farms. Chickens aged from 15 to 35 days old. The main gross lesions were perihepatitis, pericarditis, and airsacculitis. Isolation was carried out on MacConkey agar (Merck, Darmstadt, Germany) and identified based on colonial morphological characters, and Analytical Profile Index (API 20E, Bio-Merck, Martillac, France) (data not shown)	this study
*E. coli-2*	E-2/20	Mueller–Hinton agar		this study
*E. coli-3*	E-3/20	Mueller–Hinton agar		this study
*E. coli-4*	E-4/20	Mueller–Hinton agar		this study
*E. coli-5*	E-5/20	Mueller–Hinton agar		this study
*E. coli-6*	E-6/20	Mueller–Hinton agar		this study
*E. coli-7*	E-7/20	Mueller–Hinton agar		this study
*E. coli-8*	E-8/20	Mueller–Hinton agar		this study
*E. coli-9*	E-9/20	Mueller–Hinton agar		this study
*E. coli-10*	E-10/20	Mueller–Hinton agar		this study
*E. faecalis-1*	EF-1/20	Mueller–Hinton agar	Isolated from gastrointestinal tract of commercial broiler chickens suffering from *E. coli* infection (see above). Isolation was done on Citrate-Azid-Tween-Carbonate agar (Oxoid, Wesel, Germany) and identified based on biochemical tests (data not shown)	this study
*E. faecalis-2*	EF-2/20	Mueller–Hinton agar		this study
*E. faecalis-3*	EF-3/20	Mueller–Hinton agar		this study
*E. faecalis-4*	EF-4/20	Mueller–Hinton agar		this study
*E. faecalis-5*	EF-5/20	Mueller–Hinton agar		this study
*E. faecalis-6*	EF-6/20	Mueller–Hinton agar		this study
*E. faecalis-7*	EF-7/20	Mueller–Hinton agar		this study
*E. faecalis-8*	EF-8/20	Mueller–Hinton agar		this study
*E. faecalis-9*	EF-9/20	Mueller–Hinton agar		this study
*E. faecalis-10*	EF-10/20	Mueller–Hinton agar		this study

Caso agar (3.5% casein-soya, 0.3% yeast extract, 0.1% glucose, 1.5% agar).

**Table 2:** In vitro cytotoxic assay using breast cell line MCF-7 and hepatoblastoma HEPG-2 human cancer cell lines.

Tested Sample	% Viability (Means ± SEM, *n* = 6)			
	MCF-7		HEPG-2	
	10 µL/mL	100 µL/mL	10 µL/mL	100 µL/mL
Total extract	97.9 ± 2.46	31.3 ± 2.87 ***	93.4 ± 2.92	46.4 ± 1.17 ***
Prepared zinc salt	90.0 ± 2.94	43.5 ± 5.84 ***	98.2 ± 1.27	5.7 ± 1.89 ***^#^

*** indicates significant cytotoxicity effects between different concentrations (*p* < 0.0001, 0.0002, 0.0024, and 0.0001, respectively). ^#^ indicates a significant cytotoxicity effect of zinc salt (*p* < 0.0001) at 100 µL/mL on HEPG-2.
